# Individualised controlled ovarian stimulation (iCOS): maximising success rates for assisted reproductive technology patients

**DOI:** 10.1186/1477-7827-9-82

**Published:** 2011-06-21

**Authors:** Ernesto Bosch, Diego Ezcurra

**Affiliations:** 1 Instituto Valenciano de Infertilidad, Valencia, Spain; 2 Merck Serono S.A., Geneva, Switzerland

## Abstract

**Background:**

In the last two decades, pregnancy rates for patients undergoing in-vitro fertilisation (IVF) have significantly increased. Some of the major advances responsible for this improvement were the introduction of controlled ovarian stimulation (COS) for the induction of multiple follicle development, and the utilisation of mid-luteal gonadotropin-releasing hormone agonists to achieve pituitary down-regulation and full control of the cycle. As a result, a combination of a gonadotropin-releasing hormone agonist with high doses (150-450 IU/day) of recombinant follicle-stimulating hormone has become the current standard approach for ovarian stimulation. However, given the heterogeneity of patients embarking on IVF, and the fact that many different drugs can be used alone or in different combinations (generating multiple potential protocols of controlled ovarian stimulation), we consider the need to identify special populations of patients and adapt treatment protocols accordingly, and to implement a more individualised approach to COS.

**Discussion:**

Studies on mild, minimal and natural IVF cycles have yielded promising results, but have focused on fresh embryo transfers and included relatively young patient populations who generally have the potential for more favourable outcomes. The efficacy of these protocols in patients with a poorer prognosis remains to be tested. When comparing protocols for COS, it is important to think beyond current primary endpoints, and to consider the ideal quality and quantity of oocytes and embryos being produced per stimulated patient, in order to achieve a pregnancy. We should also focus on the cumulative pregnancy rate, which is based on outcomes from fresh and frozen embryos from the same cycle of stimulation. Individualised COS (iCOS) determined by the use of biomarkers to test ovarian reserve has the potential to optimise outcomes and reduce safety issues by adapting treatment protocols according to each patient's specific characteristics. As new objective endocrine, paracrine, functional and/or genetic biomarkers of response are developed, iCOS can be refined further still, and this will be a significant step towards a personalised approach for IVF.

**Conclusions:**

A variety of COS protocols have been adopted, with mixed success, but no single approach is appropriate for all patients within a given population. We suggest that treatment protocols should be adapted for individual patients through iCOS; this approach promises to be one of the first steps towards implementing personalised medicine in reproductive science.

## Background

Since the first successful IVF-embryo transfer (IVF-ET) was carried out in 1978, the treatment of infertility has advanced significantly [1]. The subsequent introduction of COS for multiple follicular development significantly increased pregnancy rates [2]. Such stimulation protocols have now been developed and refined for more than 25 years in an attempt to obtain an optimal number of oocytes from each treatment cycle, and to maximise pregnancy rates per fresh ET.

A significant milestone in the development of COS was the implementation of gonadotropin-releasing hormone (GnRH) agonists for pituitary suppression, from the mid-luteal phase of the prior cycle until the completion of the COS process (long protocol). This approach allows IVF centres to manage patients more easily, thereby reducing cycle cancellation rates (as high as 35% before the introduction of GnRH agonists) [3,4], results in greater numbers of oocytes retrieved, and produces better quality embryos and higher pregnancy rates than older protocols [5]. In current care, the GnRH agonist long protocol combined with high doses (150-450 IU/day) of recombinant follicle-stimulating hormone (r-FSH) continues to be the most commonly used COS approach [6,7], although the use of human menopausal gonadotropin (hMG) is also well established [8]. This article will discuss proposed alternatives in clinical practice to the standard long protocol, the need to adapt approaches for patients sub-populations, and finally will consider the use of biomarkers as a tool for implementing an individualised approach to COS treatment protocols, and thus move into a new era of personalised medicine.

## Discussion

### Alternative COS protocols and the need for individualised treatment

**Figure 1:**
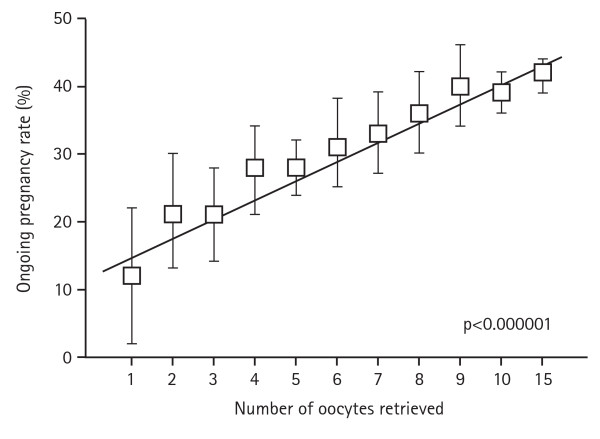
**Relationship between number of oocytes retrieved and ongoing pregnancy rate**. Ongoing pregnancy rate according to ovarian response in an unselected population (source: Instituto Valenciano de Infertilidad, between 2004 and 2008, n = 7954, p < 0.000001 [Mantel-Hansen test for trend]).

The importance of achieving a good response to COS is underscored by the fact that the number of oocytes obtained following stimulation correlates positively with the ongoing pregnancy rate (Figure 1) (data on file from an unselected population of patients from the Instituto Valenciano de Infertilidad, Valencia, Spain) [9]. However, a significant proportion of patients show a low or poor response to the classical approach of the GnRH agonist long protocol, which does not make it the ideal choice for them. Consequently, several modifications and alternatives will be discussed that have been introduced to improve outcomes. These options should be considered in the context that success rates for IVF remain low, and that further individualising treatment has the potential to produce significant improvement in pregnancy rates.

### Adjusting the standard protocols for COS

The 'short', 'ultrashort', 'micro-flare' and 'stop' GnRH agonist protocols involve adjusting the timing and dose of GnRH agonist administration, so that the patient benefits from the initial flare-up of endogenous follicle stimulating hormone (FSH) and luteinising hormone (LH) that may 'jump start' the follicles, in addition to the action of exogenous gonadotropins [10,11]. Another modification to the standard long protocol involves changing the combination of gonadotropins. For example, the addition of LH has been shown to improve cycle outcomes in poor responders and patients > 35 years old [12,13]. Moreover, pre-treatment with testosterone [14,15], oestrogens [16] or letrozole [17] has also been proposed to increase ovarian response in this particular population.

### Mild and minimal ovarian stimulation

The traditional approach of an agonist long protocol which aims for an optimal number of oocytes, is not without drawbacks. The protocol is time consuming and involves complex regimens (at least 3 weeks of daily injections) that cause considerable patient discomfort, and has important short-term complications including ovarian hyperstimulation syndrome (OHSS) and a high incidence of multiple pregnancies. These negative aspects lead to a high rate of drop-outs [18], and increased costs [19]. As a result, alternative COS approaches have been developed, whose aim is to optimise the likelihood of achieving a healthy birth at a reasonable cost, while ensuring patient comfort and reducing the incidence of complications. These mild stimulation protocols are less complex and time consuming, less expensive, have lower drop-out rates, and are hypothesised to have improved oocyte quality and endometrial receptivity than the traditional approach [20].

Mild ovarian stimulation is based on two principles: the use of GnRH antagonists, which cause immediate and dose-dependent gonadotropin suppression [21], and the concept that the impact of r-FSH on follicular recruitment depends on the length of exposure above a threshold, rather than the degree of FSH elevation [22]. This mild stimulation achieved similar pregnancy rates per started cycle compared with the conventional regimen, or compared with the standard GnRH agonist long protocol [23]. Furthermore, the use of this approach has been shown to obtain a higher proportion of chromosomally normal embryos than a conventional stimulation strategy aimed at maximising oocyte yield [24] with less psychological impact [25] and lower costs per year of treatment [26].

An even more simplistic approach to ovarian stimulation that has been proposed [27] uses clomiphene citrate as an antagonist of hypothalamic oestradiol receptors, thereby inhibiting both negative and positive feedback. Thus, in the minimal ovarian stimulation protocol, clomiphene citrate is used both to stimulate follicular development and to suppress ovulation. Pregnancy rates per transfer (34.1%) and the number of viable vitrified embryos (1.1 ± 1.5) were promising, and were significantly higher with this regimen than an adjusted regimen using a lower dose of HMG or clomiphene citrate alone, as shown in a retrospective analysis of 3654 cycles [28].

### Natural ovarian cycles

In any discussion of approaches to COS, natural and modified natural cycles should be taken into account. Natural cycles involve monitoring a patient's spontaneous cycle, and retrieving a single oocyte prior to the LH peak; data show that this approach leads to a significantly lower pregnancy rate than is achieved with stimulated cycles [9]. In modified natural cycles, a cumulative ongoing pregnancy rate of 30% after six cycles has been reported, and the protocol has been proposed as a good option before attempting COS IVF [29].

### Comparing ovarian stimulation protocols

There have been very encouraging results from studies on mild, minimal and natural ovarian stimulation approaches, in which fresh ETs were used exclusively [23,27-29]. However, the patients included in these studies were relatively young (mean age 33-34 years), lean (body mass index [BMI] between 23 and 24), with normal ovarian reserve and mostly with male, tubal or unexplained infertility, and so constitute a population in which the outcomes of fertility treatment are generally favourable. Unfortunately, the efficacy of these protocols in patients with a poorer prognosis remains uncertain.

It is also important to consider the lack of reports on the outcomes of pregnancies generated from frozen-thawed IVF cycles. When only fresh ETs are analysed, several published studies have been unable to detect a significant difference between regimens [30-32]. It is therefore inaccurate to consider only the results of stimulation protocols that are based exclusively on fresh transfers. Cumulative pregnancy rates are more meaningful, being calculated by combining the outcomes from fresh and frozen-thawed embryos from the same cycle of stimulation. In our experience, a maximum of 8-9 retrieved mature oocytes is enough to achieve the highest cumulative pregnancy rates. Any protocol of stimulation that results in an increased number of good quality oocytes per cycle will not lead to a higher chance for a patient to become pregnant.

Furthermore, when considering what a successful outcome means to a couple suffering from infertility, it is important to remember that the most relevant measure of success is the ability to have a single healthy child delivered per initiated cycle, one patient, one embryo and one baby [33]. To achieve this endpoint, it is of paramount importance to individualise COS protocols to be able to produce the optimal quantity and quality of oocytes and embryos, and thereby to maximise the chances of overall success. Without individualising treatment, the current move towards single ET is likely to have an adverse effect on pregnancy rates [34].

### How can patients and protocols be matched?

Matching patients with the ideal COS protocol is difficult because the outcome of ovarian stimulation is determined by many interacting factors (genetic and non-genetic), which influence the level of response achieved. The importance of each of these variables to the final outcome should not be underestimated; for example, the demographic characteristics of patients undergoing IVF have a crucial impact on the chances of success. Besides the well known impact of age [35] and ovarian reserve [9], the presence of endometriosis [36], polycystic ovaries [37] and a high BMI [38], can also affect ovarian response and/or cycle outcome. In reality, IVF units encounter a great variety of pathologies.

Table 1 shows the demographic characteristics of a population undergoing IVF in a large university-affiliated unit; the proportion of patients with a good prognosis was only 32%. Furthermore, in patients with the same pathologies, genetic differences may predispose some individuals to respond better to one stimulation protocol over another. Fortunately, as described previously, there are many different options for COS, based on different combinations of GnRH analogues and stimulation drugs (Table 2). However, given the factors mentioned above, it is inaccurate to put forward one single protocol for a particular patient group, when there may be considerable genetic variation within that population. At the present time, COS is generally being utilised in an empirical way; and unfortunately, trial-and-error methodology is the most frequent path for infertile patients. The type of protocol that is selected for a patient is dependent on multiple factors, including physician experience, the individual patient pathology, age, BMI and practice guidelines of individual fertility clinics.

**Table 1:** Characteristics of patients undergoing IVF in the Instituto Valenciano de Infertilidad between 2004 and 2008 (n = 7954)

Characteristics	Age group (years)					
	≤35		36-40		> 40	
BMI	< 25	≥ 25	< 25	≥ 25	< 25	≥ 25
Normo ovulatory (%)	31.9	5.6	19.3	4.1	7	1.8
Anovulation/PCOS (%)	4.5	2.5	1.4	0.9	0.06	0.04
Low responders (%)	4.4	0.7	3.6	0.6	0.34	0.06
Endometriosis (%)	5.7	0.4	2.7	0.2	0.18	0.02

**Table 2:** Choices for COS according to possible combinations of GnRH agonist/antagonist and stimulation drugs

	GnRH agonist/antagonist protocol							
	GnRH agonist			GnRH antagonist			No GnRH analogue	
	Long	Short	Micro flare	Standard	Mild	Modified natural	Mini	Natural
Gonadotropins and other agents								
FSH								
HMG								
FSH + LH								
Others: Clomiphene								
Letrozole								
Testosterone								
Oestrogens								

### Individualised COS (iCOS): the first step towards personalised medicine

The diversity seen in the population of fertility patients, means that continuing with a single approach to treatment is unlikely to further improve outcomes. Reproductive medicine is similar to other therapeutic areas in that personalised medicine and customised therapy is still under some development, and there are several barriers to overcome [39]. On one side, some pharmaceutical companies involved in the fertility field promote a *blockbuster model*, focused on developing and marketing drugs for use in as broad a patient group as possible, while discouraging the development of therapies for smaller sub-populations and the diagnostic tests that can identify those sub-populations. On the other side, regulatory agencies cause too many resources to be devoted to Phase III clinical trials, which mean that very few resources are available for post-approval drug monitoring and assessment. In addition, clinicians' daily practice is often empirical, despite the availability of diagnostic tests that could guide more personalised prescription of drugs and procedures. A further issue is that clinical trials for new drugs generally use the best population of patients to capture data against a comparator under ideal conditions. However, this population often represents only a small proportion of the patients typically seen in day-to-day clinical practice.

The adoption and expansion of iCOS through the application of a combination of biomarkers of ovarian reserve, follicle recruitment and genetic configuration of the receptors for the hormones utilised is required. Pharmacogenomics is the branch of pharmacology, which examines the influence of genetic variability on the variation in the response to drugs seen between patients. By studying correlations between gene expression or single-nucleotide polymorphisms and the efficacy or toxicity of a drug, pharmacogenomics aims to provide a rational way to optimise drug therapy according to the patient's genotype, to ensure maximum efficacy with minimal adverse effects. Such approaches promise the advent of 'personalised medicine', in which drugs and drug combinations are tailored to each individual's unique genetic make-up. As an example of the issues which this approach may help to address, the study of Shahine and colleagues has recently shown that Asian women had significantly lower implantation, clinical pregnancy, and live birth rates than Caucasian women, even though the population was composed exclusively of women in whom only the highest quality embryos had been transferred [40]. In addition, a study on FSH receptor gene polymorphisms has suggested that variation in the response to FSH is related to the fact that women with ovarian dysfunction tend to carry the Ser/Ser allelic variant, whereas good responders more often carry the Asn/Ser allelic variant, which has a higher FSH sensitivity [41]. Further work to clarify the utility of FSH receptor gene polymorphisms is needed [42].

The analysis of LH receptor polymorphisms has also produced interesting results. It has been shown that the incidence of a common LH receptor polymorphism is significantly higher in patients needing high doses of r-FSH for COS [43]. In addition, these patients have been shown to have a better outcome when recombinant LH is added to r-FSH for COS [44,45]. Therefore, this approach could be useful for determining the need for LH in a particular population undergoing COS.

### iCOS: how to customise IVF treatment using biomarkers

As previously discussed, patient age, hormonal status, PCOS, endometriosis and previous response to COS are all factors that contribute to infertility and impact on the outcome of IVF. We already treat patients differently depending on these characteristics, however, there are further clinical features that indicate patients who may benefit from COS tailored to their specific needs. It is feasible to further define every patient before starting a COS cycle, by analysing various endocrine/paracrine biomarkers, including FSH, anti-Müllerian hormone (AMH) or inhibin B hormone levels, as well as functional biomarkers such as antral follicle count (AFC) [46]. These biomarkers can provide a very specific characterisation of a particular patient and each has its own advantages (Table 3),

**Table 3:** Characteristics of potential markers for response to COS (where +++ = degree to which a characteristic is present)

Characteristics of an effective marker	Age	AMH	FSH	AFC
Prediction of poor response	+	+++	++	+++
Prediction of hyper response	+	+++	-	++
Low inter-cycle variability	+++	++	-	++
Low intra-cycle variability	+++	++	-	++
Applicable to all patients	+++	+++	+	+
Low cost of applying test	+++	-	-	-

Day 3 FSH, oestradiol and inhibin B have traditionally been used as indicators of ovarian reserve. Inhibin B is a protein produced by the granulosa cells of pre-and early-antral follicles, and circulating levels of inhibin B are highest during the earliest and mid-stages of the normal menstrual cycle [47]. FSH and oestradiol also vary during the menstrual cycle and even though these molecules were suggested as direct biomarkers of ovarian reserve over a decade ago, there is still some doubt as to the validity of this hypothesis, and further clarification is needed [46,48-51].

AFC can be used as an indicator of the number of follicles present. AFC does not change during the menstrual cycle [52] but has been shown to steadily decrease during the reproductive years. This decline in AFC is in line with the belief that the number of antral follicles indicates the size of the primordial follicle pool [53]. However, AFC is of limited clinical value for use in the prediction of pregnancy [46].

AMH is a member of the transforming growth factor-β family, and is the first predictive paracrine biomarker used to anticipate the magnitude of ovarian response in women undergoing IVF. AMH is primarily a product of the granulosa cells in the pre-antral and small-antral follicles [54] and correlates with AFC. AMH appears to be more accurate in predicting ovarian response than patient age, ovarian volume or day 3 levels of FSH, oestradiol or inhibin B [55]. An additional benefit of AMH, is that it is a stable biomarker within and between menstrual cycles [56,57], removing the need for cycle stage dependent blood samples or ultrasound scans. Serum AMH currently has the strongest predictive capability for ovarian response and pregnancy for couples with advanced female age or absence of male factors [58].

A recent review of over 20 retrospective and prospective studies of the use of AMH as a marker of ovarian response to COS showed a positive correlation between basal AMH serum levels and the number of retrieved oocytes in women undergoing ovarian stimulation [59]. There have been a limited number of studies published to date on the relationship between AMH levels and OHSS, however, studies show that hyper stimulation and OHSS may be associated with higher mean basal AMH levels [55,60-65]. The studies by Lee et al. and Nardo et al. showed that basal AMH levels above 3.5 ng/ml are good predictors of hyper response and OHSS, and patients who fall into this group may benefit from the use of milder, more patient-friendly stimulation protocols [64,65].

Further evidence of the use of AMH and AFC as predictors of hyper response in COS was provided by Broer et al. who conducted a systematic review and meta-analysis of the existing literature. The authors concluded that AMH and AFC levels could potentially be used to individualise FSH dosing regimens during COS [66]. It is important to note that AMH levels can also be useful in identifying oocyte donors who are at risk of developing OHSS, and assist in appropriate dose adjustment [67].

There have been very few studies that have investigated the relationship between serum AMH levels following IVF, and the number of live births achieved. Nelson et al., 2007 demonstrated in a prospective study of 340 women that the live birth rate increased with increasing basal AMH levels. However, this was only seen in women with basal AMH levels < 7.8 pmol/L [55].

It is likely that AMH cannot predict if a patient will become pregnant, but that it can predict patients who have a higher probability of becoming pregnant following IVF, as well as identifying those at higher risk of developing OHSS.

In addition to only looking at AMH levels and outcome of IVF, a recent prospective cohort study of 538 women by Nelson et al., 2009 went a step further and investigated the relationship between AMH levels and the success of different IVF treatment protocols. Women with high AMH levels (> 15 pmol/L), classified as high responders, who had a low starting dose of FSH followed by a GnRH antagonist protocol rather than an agonist protocol, eliminated the need for complete cryopreservation of embryos due to excess response. These women also had a higher fresh cycle clinical pregnancy rate and fewer hospitalisations for OHSS. Women with normal AMH levels (5-15 pmol/L) treated with a traditional GnRH agonist long protocol, showed a low incidence of excess response and poor response. Finally, women with low AMH (1- < 5 pmol/L), classified as reduced responders, exhibited a suboptimal response to COS and low pregnancy rates irrespective of the treatment strategy used [68].

In addition to the hormone and functional biomarkers mentioned, genetic traits and abnormalities can also influence fertility and could provide further biomarkers. Identification and in vitro characterisation of four abnormal FSH receptor variants indicates that screening of patients before embarking on stimulation with FSH may be beneficial for success [69]. It has also been suggested that hyposensitivity to FSH may be caused by an abnormal variant of the FSH receptor that reduces sensitivity to the receptor [70]. Mutations in the genes coding for LH [71-75] and the LH receptor [76-78] have been identified and these mutations may play a role in the cause of infertility, as well as influence the success or failure of fertility treatment. Further, it has been suggested that assessing serum androgen levels prior to initiation of COS may be beneficial for the selection of gonadotropin starting doses. An assessment of theca cell function following a GnRH agonist stimulation test prior to the initiation COS revealed that serum steroid levels correlated with AFC, as well as with sensitivity to FSH [79].

There is also currently a large body of work underway to find novel and objective biomarkers for oocyte and embryo quality, and for other factors such as endometrial status, [58,80,81], which in the future may offer the possibility of refining IVF even further. Further large-scale studies to determine the relationship between levels of biomarker such as AMH and the success of different IVF treatment protocols in different patient populations are required to fully realise the future potential of IVF success.

### The future potential of increased IVF success through the development and implementation of iCOS

Today, after 30 years of IVF practice, the live birth rate for patients with a very good prognosis is still below 50%. Taking a qualitative leap in IVF techniques, by introducing a more individualised approach such as iCOS could dramatically improve results and reduce safety issues. We must match the right drugs and protocols of stimulation to the right patients. By developing technologies to improve culture conditions and to objectively identify the gametes that will generate embryos with high implantation potential, the extent of the improvements that iCOS can facilitate is almost unimaginable. To complete the circle, if we develop technologies that allow us to define endometrial receptivity status, we will be able to revolutionise the system, and therefore achieve the best outcomes for the benefit of our patients.

We foresee a future in which patients will be tested to define their endocrine/paracrine status and genetic make-up to define a path for iCOS that will be adapted according to their individual needs. The iCOS protocols will be designed to produce an ideal number of high quality oocytes and embryos, and advanced technologies will allow us to select the optimal embryo to transfer. Single ET, vitrification and objective uterine receptivity tests will maximise each patient's chance of achieving a successful pregnancy.

## Conclusions

The introduction of COS to IVF approaches has significantly improved outcomes, but current stimulation protocols are not optimal for all patient groups. In addition, COS regimens are complex and may have negative effects such as OHSS. Alternatives to standard COS protocols, including mild and natural cycles, have shown some success, but no single approach is appropriate for all patients in a given population. We propose that treatment should be adapted for individual patients through iCOS and that, together with the further development of objective biomarkers of response, will be an important first step towards implementing personalised medicine in reproductive science.

## List of abbreviations

AFC: antral follicle count; AMH: anti-Müllerian hormone; BMI: body mass index; COS: controlled ovarian stimulation; FSH: follicle-stimulating hormone; GnRH: gonadotropin-releasing hormone; HMG: human menopausal gonadotropin; iCOS: individualised controlled ovarian stimulation; IVF-ET: in-vitro fertilisation-embryo transfer; LH: luteinising hormone; OHSS: ovarian hyperstimulation syndrome; PCOS: polycystic ovary syndrome; r-FSH: recombinant follicle-stimulating hormone.

## Competing interests

EB has received consultation fees from Merck-Serono and Schering Plough, and honoraria for participating in sponsored symposiums by Merck-Serono S.A.-Geneva, Schering Plough and Ferring Pharmaceuticals. DE is an employee of Merck Serono S.A.-Geneva, Switzerland (an affiliate of Merck KGaA, Darmstadt, Germany).

## Authors' contributions

EB and DE agreed the scope of this article, prepared the initial draft, and reviewed the manuscript at all stages. They are fully and equally responsible for the content of the article. Both authors read and approved the final manuscript.
